# Association between high serum ferritin and periodontitis: A population‐based cross‐sectional preliminary study

**DOI:** 10.1002/JPER.24-0491

**Published:** 2025-06-10

**Authors:** Susilena Arouche Costa, Cecilia Cláudia Costa Ribeiro, Ana Regina Oliveira Moreira, Gustavo G. Nascimento, Soraia de Fátima Carvalho Souza

**Affiliations:** ^1^ Postgraduate Program in Dentistry Federal University of Maranhão São Luís Maranhão Brazil; ^2^ National Dental Research Institute Singapore National Dental Centre Singapore Singapore Singapore; ^3^ Oral Health Academic Clinical Program Duke‐NUS Medical School Singapore Singapore

**Keywords:** ferritin, inflammation, iron, periodontal disease, periodontitis

## Abstract

**Background:**

The aim of this study was to investigate the association between high serum ferritin levels and periodontitis considering pathways induced by sociodemographic and behavioral factors, serum inflammation, and metabolic risk.

**Methods:**

Data from 7283 individuals who participated in the Third National Health and Nutrition Examination Survey (NHANES III) were analyzed. We estimated indirect and direct pathways for the association between high serum ferritin levels (in quintiles) and periodontitis. A theoretical model was constructed to evaluate the association between high serum ferritin and periodontitis, considering direct and indirect (mediated) pathways, including poverty, age, smoking, alcohol consumption, obesity, iron consumption, serum inflammation (C‐reactive protein [CRP]), and insulin resistance (Homeostatic Model Assessment of Insulin Resistance [HOMA‐IR]), analyzing through structural equation modeling (SEM).

**Results:**

Higher ferritin levels were directly associated with periodontitis (standardized coefficient [SC] = 0.074, standardized error [SE] = 0.033, *p*‐value = 0.025). Furthermore, higher ferritin mediated the association between serum inflammation (SC = 0.012, SE = 0.005, *p*‐value = 0.033) and periodontitis.

**Conclusion:**

Higher ferritin levels may play a role in periodontitis as part of a systemic inflammation mechanism.

**Plain Language Summary:**

Mechanisms that justify the association between increased ferritin and periodontitis are still poorly understood. Ferritin is the main marker of iron stores, but it can be influenced by several factors that are also related to periodontitis. Our study investigated indirect (inflammatory) and direct (local) mechanisms for the association between ferritin and periodontitis. The systemic inflammatory mechanism partially explained the association between ferritin and periodontitis. Elevated ferritin levels remained directly associated with periodontitis even after adjustment for confounding, indicating the possibility of a direct mechanism. Elevated levels of ferritin may contribute to the severity and extent of periodontitis.

## 1 INTRODUCTION

Iron is essential for various biological processes in the human body, such as oxygen transport, DNA synthesis, and energy production; however, it can also generate reactive oxygen species (ROS), causing oxidative damage.^1^ Commonly used to assess iron stores in the body, increased ferritin levels have been linked to several chronic diseases,^2^, ^3^ including periodontitis in specific populations such as women with anorexia nervosa,^4^ in postmenopausal women,^5^ and in diabetic patients.^6^

In these specific populations, the direct mechanism is the primary mechanism used to explain the relationship between high ferritin levels and periodontitis. This considers iron's role in bacterial growth and regulating periodontal pathogens such as *Porphyromonas gingivalis*, *Treponema denticola*, and *Prevotella intermedia*.^7^, ^8^ Iron also influences stem cell differentiation in the human periodontal ligament and regulates the host's innate immune system.^9^ Beyond this direct mechanism, various factors can influence ferritin synthesis and impact periodontal tissue breakdown in both men and women from the general population, highlighting the need for further investigation into this association.

For example, unhealthy lifestyle behaviors may play a role in this process; for instance, alcohol abuse and smoking can both lead to higher ferritin levels ^10^, ^11^ and more severe periodontitis.^12^, ^13^ Moreover, a diet high in iron, particularly excessive consumption of red meat (heme‐iron), is a significant predictor of increased iron levels in the body;^14^ it also has been implicated in greater probing depth (PD) and bleeding on probing.^15^

Furthermore, metabolic factors may significantly influence the association between ferritin and periodontitis. Obesity and low‐grade systemic inflammation contribute to the severity of periodontitis,^16^, ^17^ and influence the body's ferritin levels.^18^ Studies have demonstrated that elevated ferritin levels can damage hepatocytes and pancreatic *β* cells, resulting in insulin resistance,^19^ a recognized metabolic risk factor for periodontitis.^20^

Therefore, we examined the relationship between ferritin and periodontitis, focusing on indirect and direct pathways through which elevated ferritin levels might be associated with periodontitis in both men and women from the general population. We hypothesized that ferritin's role in the destruction of periodontal tissues might occur through (1) inflammatory mechanisms associated with behavioral and metabolic risk factors, (2) increased insulin resistance, or (3) a direct mechanism.

## 2 MATERIALS AND METHODS

We analyzed data from the Third National Health and Nutrition Examination Survey (NHANES III), conducted in the United States from October 1988 to October 1994. NHANES III is a cross‐sectional study that used complex, multi‐stage sampling, stratified representative of non‐institutionalized Americans. All participants were interviewed using structured questionnaires that included demographic, socioeconomic, smoking, and alcohol abuse, among others. Participants also underwent medical, dental, and laboratory examinations at the mobile examination center. During the survey, 39,695 people were selected over the 6 years; of these, 33,994 were interviewed at home and 14,421 underwent a complete periodontal examination.

Only individuals under 50 years of age who have undergone periodontal examination were considered eligible for the present study, since climacteric women (> 50) and the elderly have altered ferritin levels^21^ and increased risk of periodontitis.^22^

### 2.1 Periodontal examination

Trained dentists performed the periodontal examination according to the NHANES examination protocol. A periodontal examination was performed in 2 quadrants (1 upper and 1 lower), which were randomly selected at the beginning of the examination. Each tooth's buccal and mesiobuccal surfaces, except the third molars, were probed separately for each periodontal clinical parameter. Thus, the PD, distance from the free gingival margin to the bottom of the sulcus/pocket, and the clinical attachment loss (CAL), the difference between the (1) recorded distance from the free gingival margin to the cementoenamel junction and (2) recorded distance from the free gingival margin to the base of the sulcus, were evaluated at 28 sites.

Periodontitis stage was classified according to the consensus proposed in the latest World Workshop on the Classification of Periodontal and Peri‐Implant Diseases and Conditions in 2017.^23^ A  periodontitis case was defined when: (1) interproximal CAL was detected in 2 or more non‐adjacent teeth, or (2) 2 or more teeth were detected with a vestibular site affected by CAL ≥ 3 mm and PD > 3 mm (in the same location).^23^

After defining the case, the periodontitis stages were classified. Those individuals who had a diagnosis of periodontitis and: (1) CAL between 1–2 mm were classified as Stage 1; (2) CAL between 3 and 4 were classified as Stage 2; and (3) CAL ≥ 5 mm were classified as Stage 3.^23^, ^24^ Generalized and localized subclassification was also used for stage 3; if < 30% of teeth were at the most severe stage, they were classified as localized; otherwise, as generalized.^23^

### 2.2 Serum ferritin levels

Blood samples were obtained at the testing center. Ferritin levels (µg/mL) were categorized into quintiles.

### 2.3 Covariables

Age (in years) and education level (categorized as ≤ 8, 9–12, and > 12 years) were obtained through interviews with structured questionnaires. The Poverty Index (categorized as ≤ 1.3, 1.4–3.5, and > 3.5) was calculated by dividing household income by specific poverty guidelines for household size, as well as the appropriate year and state.

Smoking status was derived from 2 questions: “Do you smoke cigarettes now?” and “Have you smoked at least 100 cigarettes in your entire life?” Thus, individuals were categorized as: (0) never smoked (when they answered “No” to both questions), (1) former smokers (for those who answered “No” to the first question and “Yes” to the second question) and (2) current smokers (when they answered “Yes” to both questions).^25^

The number of days that alcohol was consumed during the previous year and the average number of alcoholic beverages that were consumed on those days were used to assess alcohol consumption. In this way, we calculated the average number of alcoholic beverages consumed per day during the previous year and categorized alcohol consumption into: (1) none, which included participants who did not consume alcohol or who consumed less than 12 drinks in the last 12 months, (2) light (≤1 drink/day), (3) moderate (1–2 drinks/day), and (4) heavy (>2 drinks/day).^26^

Diet was assessed using a food recall, in which the types and amounts of all foods and beverages consumed by the participant in the last 24 h were collected. The total amount of iron ingested in the diet, categorized into quintiles, was calculated from information from the U.S. Department of Agriculture's Food and Nutrient Database.

Obesity was assessed using the body mass index (BMI) and categorized as (0) non‐obese, when BMI < 30; and (1) obese, when BMI ≥ 30^27^. The insulin resistance index (Homeostatic Model Assessment of Insulin Resistance [Homa‐IR]) was calculated from the formula insulin (uU/mL) × blood glucose (mmol/L)/22.5^28^ ^,^ and the 95th percentile was used to dichotomize the variable. C‐reactive protein, used to assess low‐grade systemic inflammation, was categorized as (0) normal when < 1 mg/dL and (1) elevated when ≥1 mg/dL.^29^ Detailed information on each specific covariate is available in the NHANES III Reference Manuals and Reports.

### 2.4 Statistical analyses

Weighted absolute and relative frequencies for categorical variables as well as mean and standard error (SE) for numerical variables were estimated.

**FIGURE 1:**
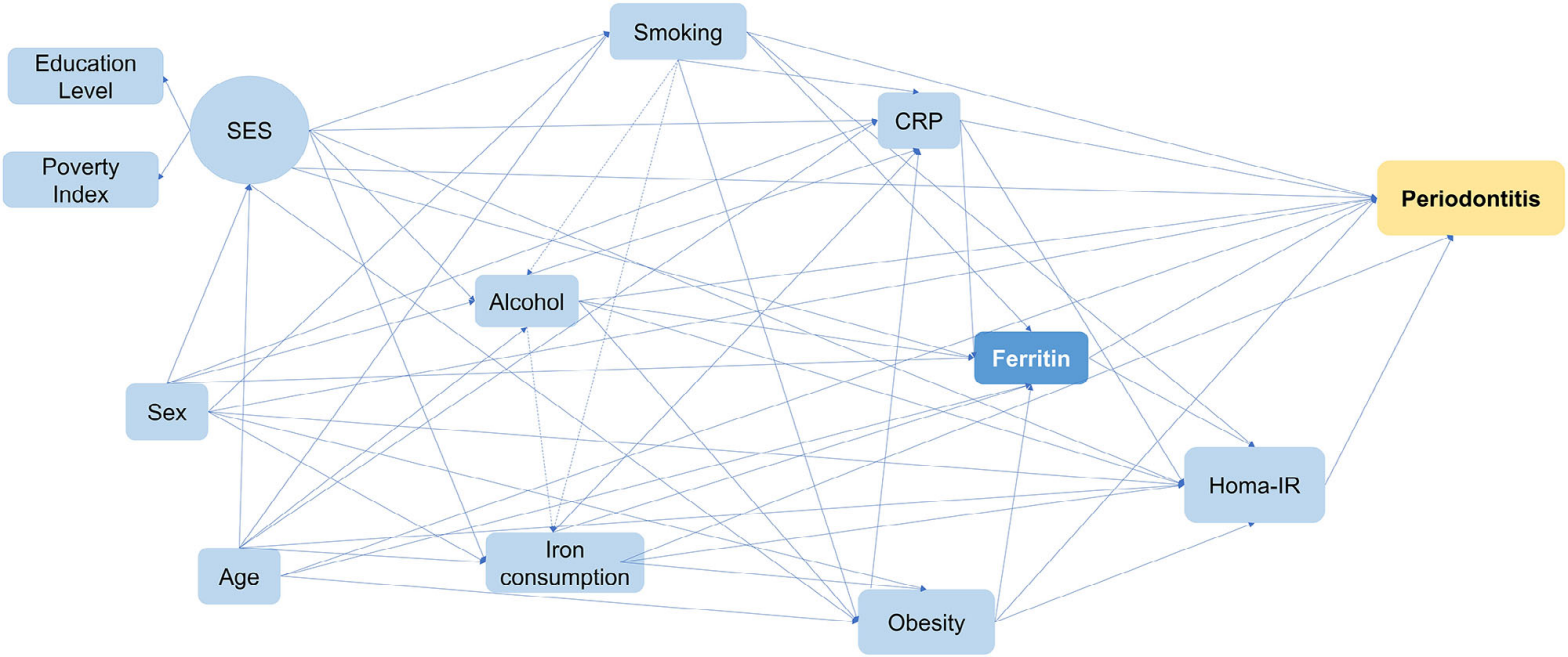
Proposed theoretical model analyzing the association between ferritin levels and periodontitis. In this model, SES is a latent variable, derived from the correlation between 2 key indicators: years of education and the Poverty Index. SES, along with age and sex, are identified as the most distal determinants influencing the other variables within the model. The model suggests that factors such as high dietary iron consumption, smoking, alcohol intake, low‐grade systemic inflammation (as indicated by CRP levels), and obesity may exert both direct and indirect effects on periodontitis through elevated ferritin levels. Additionally, ferritin is posited to influence periodontitis directly or indirectly by insulin resistance (HOMA‐IR). In the sensitivity analyses, the outcome variable for “periodontitis” was operationalized using alternative definitions: CAL ≥ 5 mm and PPD ≥ 4 mm, allowing for a more comprehensive understanding of periodontal disease severity. CAL, clinical attachment level; CRP, C‐reactive protein; HOMA‐IR, Homeostatic Model Assessment of Insulin Resistance; PPD, probing pocket depth; SES, socioeconomic status.

Based on a theoretical model proposed for the study (Figure 1), we analyzed the mechanisms for the association between ferritin and periodontitis using structural equation modeling (SEM). SEM is a statistical approach that models complex relations among a set of variables, allowing effect decomposition and explicitly identifying direct and mediated relationships.^30^ SEM also allows us to estimate latent variables deduced from the correlation among indicator variables, representing a shared variance of phenomena to reduce measurement errors.^31^

Confirmatory factor analysis was used to assess the convergence of the latent variable Socioeconomic Status, assuming a factor loading > 0.30 and *p* < 0.05. To assess the goodness of fit, the following parameters were adopted: (a) Root Mean Square Error of Approximation (RMSEA) *p* > 0.05 and an upper limit of the 90% confidence interval below 0.08; (b) Comparative Fit Index (CFI) and Tucker–Lewis Index (TLI) > 0.90. In SEM, the weighted least squaress (WLSM) estimator and theta parameterization were used.

To analyze the consistency of our results, we performed a sensitivity analysis considering the extent of periodontitis as an outcome. The extent of periodontitis was assessed by (1) proportion of number of sites affected by CAL ≥ 5 mm/total number of probed sites, and (2) proportion of number of sites of sites affected by PD≥4 mm/total number of sites probed. Furthermore, a sensitivity analysis, considering bidirectional relationships between CRP and ferritin, HOMA‐IR, and periodontitis stages, was also performed (see Figures S1 and S2 in the online *Journal of Periodontology*). To better isolate the effects of ferritin levels on periodontitis without confounding the influence of some chronic diseases, we also excluded individuals with self‐reported diabetes and cardiovascular diseases, as well as altered alanine and aspartate serum levels (*n* = 6620).

As instructed in the NHANES Manual for Statistical Analysis, all analyses performed considered the study's complex sampling, sample weight, clusters, and stratums to produce generalizable estimates for the North American population.

## 3 RESULTS

Among the subjects included in this study, 50.19% were women, 31.78% were smokers, and 19.44% were obese. Alcohol consumption was considered heavy in 4.44% of the sample. The mean (SE) of serum ferritin levels was 112.62 µg/mL (3.17). Approximately 8% of the sample had Stage 3 periodontitis (Table 1).

**TABLE 1:** Descriptive analysis of adult Americans, NHANES III (*n* = 7283).

Variables	*n*	%^a^
**Sex**		
Male	3361	49.81
Female	3922	50.19
**Level of education**		
≤8 years	1067	6.215
9 to 12 years	3932	49.41
> 12 years	2284	44.37
**Poverty index**		
≤1.3	2375	17.77
1.4 a 3.5	2953	44.09
>3.5	1955	38.14
**Smoking**		
Never	4094	49.81
Former	1092	18.41
Current	2097	31.78
**Alcohol consumption**		
Never	3422	38.56
<1 drink/day	3105	50.46
1‐2 drinks/day	411	6.68
>2 drinks/day	345	4.3
**C‐Reactive protein**		
<1 mg/dL	6667	93.39
>1 mg/dL	616	6.60
**Obesity**		
No	5,531	80.56
Yes	1752	19.44
**Periodontitis stages**		
No periodontitis	581	7.60
Stage I	4385	61.28
Stage II	1638	22.77
Stage III (localized)	609	7.38
Stage III (generalized)	70	0.95

	Mean	SE^b^
Age	32.61	0.23
Ferritin	112.62	3.17
HOMA‐IR	632.28	417.02

Abbreviation: HOMA‐IR, Homeostatic Model Assessment of Insulin Resistance; NHANES III, Third National Health and Nutrition Examination Survey.

^a^ Weighted percentage values.

^b^ SE = standard error.

The final models had a satisfactory fit for all indices used: stages of periodontitis (RMSEA = 0.011, 90% CI: 0.000–0.020, *p*‐value > 0.05, CFI = 0.99 and TLI = 0.98), proportion of sites affected by CAL ≥ 5 mm (RMSEA = 0.011, 90% CI: 0.000–0.019, *p*‐value > 0.05, CFI = 0.99 and TLI = 0.98), and proportion of sites affected by PD≥4 mm (RMSEA = 0.010, 90% CI: 0.000–0.019, *p*‐value > 0.05, CFI = 0.99 and TLI = 0.98]. The indicators of the latent variable Socioeconomic Status presented factor loadings of 0.68 (*p* < 0.001) for the variable years of education, and 0.60 (*p* < 0.001) for the variable Poverty Index, suggesting convergent validity of the construct.

**FIGURE 2:**
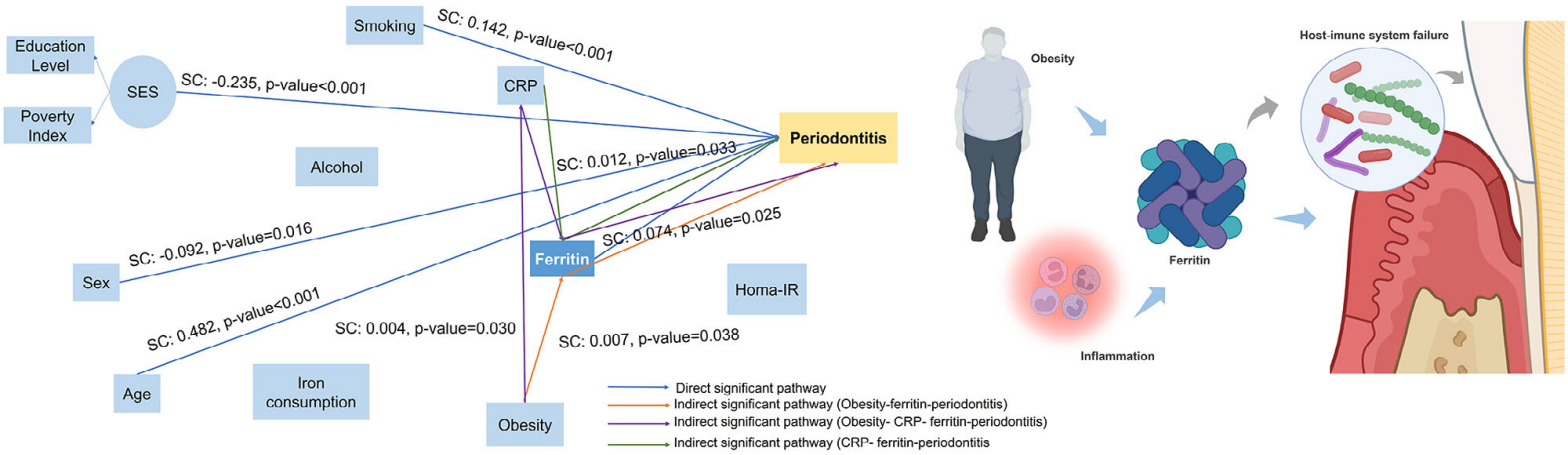
Main results from principal Structural Equation Model analysis. High ferritin levels were directly associated with more advanced stages of periodontitis; probably, this result can be justified by failure in the host's immune system, reducing the protective response against periodontopathogens. Additionally, ferritin was a mediator in the association between low‐grade systemic inflammation and Periodontitis Stages. Higher socioeconomic status and female sex were associated with lower periodontitis stage, indicating a protective effect. Smoking and age showed positive associations with higher periodontitis stages, indicating increased severity. Only direct and indirect significant pathways are shown. CRP, C‐reactive protein; SC, standardized coefficient; SES, socioeconomic status.

In the final structured model, high levels of ferritin were directly associated with Periodontitis Stages (standardized coefficient (SC) = 0.074, SE = 0.033, *p*‐value = 0.025). Ferritin was a mediator in the association between low‐grade systemic inflammation and Periodontitis Stages (SC = 0.012, SE = 0.005, *p*‐value = 0.033). Higher socioeconomic status (SC = ‐0.235, SE = 0.033, *p*‐value < 0.001) and being female (SC = −0.092, SE = 0.032, *p*‐value = 0.016) were associated with lower outcome values, while smoking (SC = 0.142, SE = 0.023, *p*‐value < 0.001) and age (SC = 0.482, SE = 0.028, *p*‐value < 0.001) were associated with higher Periodontitis Stages (Figure 2).

**FIGURE 3:**
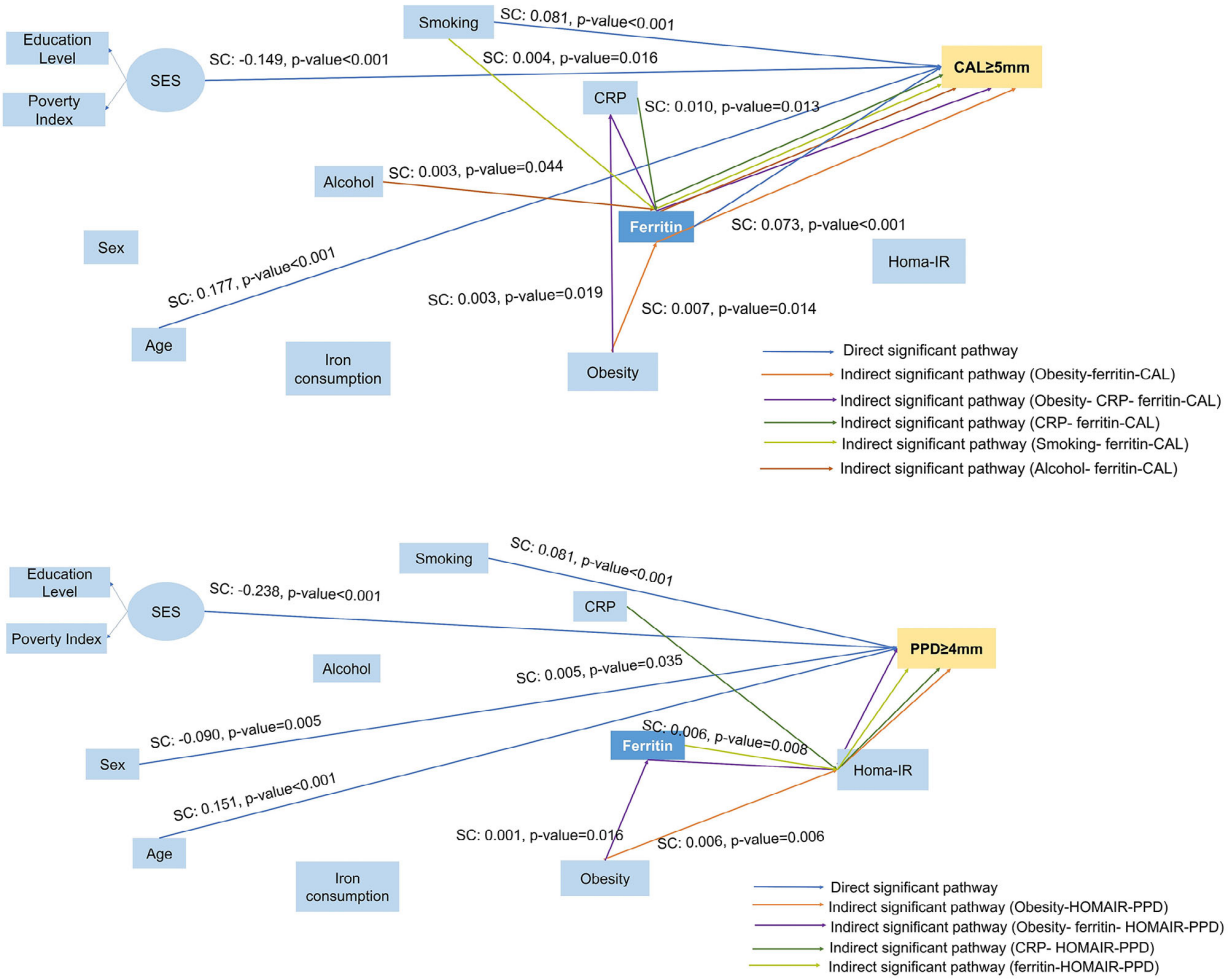
Results from sensitivity analyses. High ferritin levels were associated with the proportion of sites affected by CAL ≥ 5 mm. Additionally, High ferritin levels was a mediator in the association between low‐grade systemic inflammation and CAL ≥ 5 mm. Furthermore, smoking status is also directly associated with a higher proportion of sites affected by CAL ≥ 5 mm, highlighting the multifactorial influences on periodontal disease severity. Only direct and indirect significant pathways are shown. CAL, clinical attachment level; CRP, C ‐reactive protein; SES, socioeconomic status; SC, standardized coefficient.

In the sensitivity analyses, high ferritin levels were directly associated with a higher proportion of sites affected by CAL ≥ 5 mm (SC = 0.073, SE = 0.020, *p*‐value < 0.001). Ferritin was a mediator in the association between low‐grade systemic inflammation and the proportion of sites affected by CAL ≥ 5 mm (SC = 0.010, SE = 0.004, *p*‐value = 0.013). Smoking was directly associated with the proportion of sites affected by CAL ≥ 5 mm (SC = 0.081, SE = 0.014, *p*‐value < 0.001) (Figure 3).

Some pathways with small effects were observed in both models. For example, ferritin was a mediator in the association between obesity and Periodontitis Stages (SC = 0.007, SE = 0.003, *p*‐value = 0.038) and between obesity and a higher proportion of sites affected by CAL ≥ 5 mm (SC = 0.007, SE = 0.003, *p*‐value = 0.014). Ferritin was also a mediator between higher alcohol consumption (SC = 0.003, SE = 0.001, *p*‐value = 0.044) and smoking (SC = 0.004, SE = 0.002, *p*‐value = 0.016), with a higher proportion of sites affected by CAL ≥ 5 mm. Ferritin was associated with a higher proportion of sites affected by PD≥4 mm, via insulin resistance (SC = 0.006, SE = 0.002, *p*‐value = 0.008). The main results are not changed by the exclusion of participants with diabetes, cardiovascular diseases, or altered alanine and aspartate serum levels (see Figure S3 in the online *Journal of Periodontology*).

## 4 DISCUSSION

Our findings indicate that higher serum ferritin levels are linked to periodontitis through indirect and direct pathways. Ferritin appears to mediate the association between serum inflammation with periodontitis, suggesting that systemic inflammation may partly account for the links identified in this study. However, the direct association observed between high ferritin levels and periodontitis, even adjusting for common risk factors, suggesting the excess of iron could contribute to the deterioration of periodontal tissues. Sensitivity analyses yielded similar results, which further confirms the reliability of our findings, showing higher ferritin levels associated with greater extension of periodontitis. Therefore, this is the first population‐based study to identify both direct and mediating pathways for the association between ferritin and periodontitis.

High ferritin was associated with low‐grade inflammation (measured by CRP) and periodontitis and with a higher proportion of sites with CAL ≥ 5 mm. This suggests that inflammatory processes may exacerbate periodontitis severity and extent in part through elevated ferritin levels, which has been observed in previous studies linking hyperferritinemia to chronic inflammatory responses.^32^, ^33^ The relationship between low‐grade systemic inflammation and ferritin is pointed out in the literature, although the direction of this relationship is still a matter of discussion.^34^ Ferritin appears to be sensitive to both acute and chronic inflammatory conditions, acting as an acute‐phase reactive protein,^33^ and could be elevated in chronic inflammatory conditions without necessarily marking high levels of iron in the body.^18^ Our sensitivity analysis revealed a correlation between CRP and ferritin, highlighting bidirectional connections. Thus, inflammation may elevate CRP levels, which in turn may influence iron metabolism and ferritin levels. Our results are justifiable, as high levels of systemic inflammatory markers seem to underlie the progression of periodontitis, being perceived even in the early stages of periodontal disease.^16^

Although some pathways have shown small effects, they also reinforce the possibility of a systemic inflammatory mechanism. For example, high ferritin was a mediator for the association of smoking, alcohol consumption, and obesity with periodontitis and with a higher proportion of sites with CAL ≥ 5 mm. As an explanation, there is a significant increase in the production and release of ferritin in obese individuals,^18^ particularly due to the heightened sensitivity of ferritin to systemic inflammation induced by adipose tissue.^18^ Adipose tissue secretes several bioactive substances, including TNF‐α, that result in endotoxin‐induced injury in various organs, including periodontal tissues.^17^ Smoking and excessive alcohol consumption seem to be determinants of systemic inflammation ^35^ and, periodontitis ^12^, ^13^. Exposure to tobacco and alcohol may increase systemic inflammation via iron metabolism.^36^, ^37^, ^38^ We also observed an indirect association between high ferritin levels and a higher proportion of sites with PD ≥ 4 mm via insulin resistance. The relationship between increased serum ferritin levels and insulin resistance has been shown by population‐based studies.^19^, ^39^, ^40^ Some researchers have pointed out that iron may have an inhibitory role in the production and secretion of adiponectin, an insulin‐sensitizing adipokine considered a specific marker of dysfunction of adipose tissue, and associated with early signs of diabetes.^41^, ^42^ However, the mediated effects observed, particularly through insulin resistance, were relatively small, and their practical clinical significance should be interpreted with caution.

Systemic inflammatory mechanisms were not the only pathways identified in our study. Higher ferritin levels were associated with periodontitis and the greater proportion of sites with CAL ≥ 5 mm, regardless of the adjustment variables used in the model. Considering that optimal iron levels appear to be essential for the differentiation of periodontal ligament cells^9^ and that changes in the dynamics of the host subgingival microbiota are observed in situations where iron is more bioavailable,^43^ we might speculate that the increase in iron stores may directly contribute to the greater degradation of periodontal tissues.

The increased bioavailability of iron in the crevicular fluid may facilitate the growth and reproduction of periodontopathogens.^7^, ^8^ Periodontopathogens such as *Porphyromonas gingivalis*, *Treponema denticola*, and *Prevotella intermedia* ^7^, ^8^ can cleave iron to induce their own growth and reproduction. It has been shown that iron acquisition was abundantly overrepresented in the microbial community of periodontitis samples and has pointed to a functional signature characteristic of periodontitis. Iron is an essential enzymatic cofactor, and microbial pathogens have developed many strategies to acquire it, inducing the healthy microbial community to transform toward a pathogenic state that gives rise to periodontitis.^43^ In addition, increased iron levels also affect the host's immune system, reducing the efficiency of monocytes, macrophages, and natural killer cells.^44^ This failure in the host's Immune System can reduce the protective response against periodontopathogens.^44^ Finally, we highlight that the increase in iron levels contributes to the catalysis of cellular reactions that result in increased production of ROS, increasing local oxidative stress;^45^ the biological effects of small and large shifts in ROS production may lead to tissue damage secondary to the induction of a pro‐inflammatory state or to direct periodontal tissue damage.^46^

As secondary results, we observed direct associations between sex, age, socioeconomic status, and smoking with periodontitis, corroborating the current scientific consensus.^12^, ^47^, ^48^ The observed disparities between the sexes may stem from several factors, including hormonal influences and lifestyle behaviors. Hormonal fluctuations, particularly estrogen, are known to affect iron metabolism and inflammatory responses.^49^ Additionally, lifestyle behaviors such as dietary patterns,^50^ smoking,^51^ and healthcare‐seeking patterns ^52^ may differ between sexes, influencing overall health and susceptibility to periodontal disease. Low educational attainment and poverty are closely interrelated and are key components of socioeconomic status, both recognized as strong social determinants of health. Higher education levels are generally associated with better health literacy, access to healthcare resources, and engagement in preventive health behaviors, all of which can contribute to improved oral health outcomes. Conversely, individuals with lower socioeconomic status may face barriers to accessing dental care and a healthy diet, leading to delayed diagnoses and treatment of periodontal disease.^53^

Unexpectedly, the total amount of iron ingested in the diet (heme and non‐heme iron) was not associated with ferritin levels. The inhibitory effect of some nutrients, such as cereals and phytates found in whole grains and legumes, can inhibit iron absorption and form insoluble complexes, contributing to this result.^10^ Similarly, calcium and polyphenols from tea and coffee can also interfere with iron absorption, which may lead to lower ferritin levels despite adequate dietary iron intake.^54^ However, we cannot rule out that the increase in ferritin levels could be attributed to an unhealthy dietary pattern with a higher inflammatory potential. Additionally, the reliance on a 24‐h dietary recall method in our study presents limitations that could impact the findings.^55^ This approach may not accurately capture the habitual dietary patterns of participants or account for variations in nutrient intake over time.^55^ Thus, further studies are needed to better assess the role of dietary iron in this association.

As a limitation, due to the design study, the temporality of the observed associations between ferritin and periodontitis cannot be determined and no causal inference can be made. However, we emphasize that cross‐sectional studies can raise new hypotheses that must be further evaluated by longitudinal studies. The need for longitudinal research is particularly important in this context, as it would allow for a better understanding of the causal pathways and the potential mechanisms underlying the observed relationships. Furthermore, as the periodontal parameters were evaluated only in 2 quadrants, the prevalence of periodontitis and the associations investigated may have been underestimated in this study.^56^ This limitation raises concerns about the internal validity of our findings, as it may not accurately reflect the periodontal status of the participants. Also, we could not exclude individuals with high iron levels due to conditions such as hemochromatosis or sickle cell anemia, as this information is confidential and unavailable in the publicly accessible database. Additionally, this study did not evaluate hormone levels and medications that influence iron levels. To minimize potential biases, however, participants over 50 years old were excluded due to the significant influence of age‐related hormonal changes and the increased likelihood of medication use, which could affect iron levels. Moreover, a significant relationship between ferritin and periodontitis was already shown in post‐menopausal women in the same dataset ^5^.

We highlight the representative sample as this study's strength, allowing us to generalize our results to the American population. In addition, the analysis using structural equation models allowed us to test direct and indirect paths, concomitantly adjusting for several common causal factors, reducing confounding bias. Finally, the robustness of our results was also verified through sensitivity analysis by using the extent of periodontitis as an outcome.

## 5 CONCLUSION

According to our findings, exposure to common causal factors that driving systemic inflammation and insulin resistance, as well as the effects of ferritin on the host's immune response, could be the pathways that may explain the association between high levels of ferritin and greater severity and extent of periodontitis.

## AUTHOR CONTRIBUTIONS

*Conceptualization; Methodology; formal analysis; writing original draft preparation; read and agreed to the published version of the manuscript*: **Susilena Arouche Costa**. *Formal analysis; visualization; editing original draft; read and agreed to the published version of the manuscript*: **Cecilia Claudia Costa Ribeiro**. *Formal analysis; visualization; editing original draft; read and agreed to the published version of the manuscript*: **Ana Regina Oliveira Moreira**. *Editing original draft preparation; read and agreed to the published version of the manuscript*: **Gustavo G. Nascimento**. *Conceptualization; Methodology; review and editing; read and agreed to the published version of the manuscript*: **Soraia de Fátima Carvalho Souza**.

## CONFLICT OF INTEREST STATEMENT

The authors have stated explicitly that there are no conflicts of interest in connection with this article.

## Supporting information



Supporting Information

## Data Availability

The data that supported this study are available in Centers for Diseases Control and Prevention at https://wwwn.cdc.gov/nchs/nhanes/Default.aspx.
