# Supplementary material for: Association between high serum ferritin and periodontitis: A population‐based cross‐sectional preliminary study
Source: J Periodontol. 2025 Jun 10;97(1):127–36. doi: 10.1002/JPER.24-0491 (PMC12902711; doi:10.1002/JPER.24-0491)
Supplement: Supplementary file 1 — Supporting Information [file JPER-97-127-s001.docx]

**Supplemental Material**

**
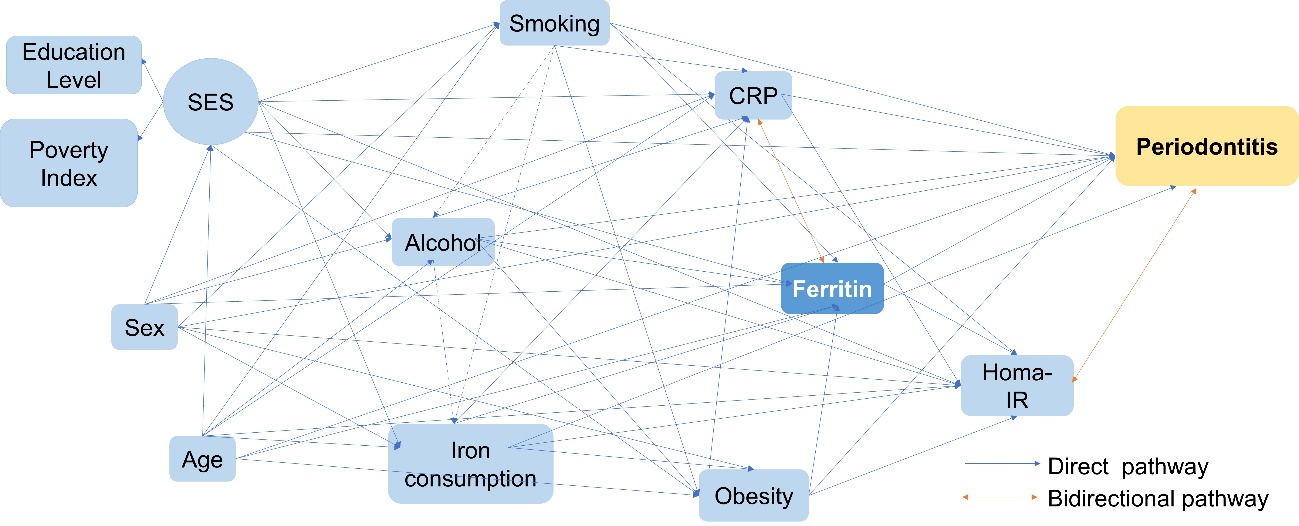
**

**Figure S1. Proposed theoretical model analyzing the association between ferritin levels and periodontitis**. In this model, Socioeconomic Status (SES) is a latent variable, derived from the correlation between two key indicators: years of education and the Poverty Index. SES, along with age and sex, are identified as the most distal determinants influencing the other variables within the model. The model suggests that factors such as high dietary iron consumption, smoking, alcohol intake, low-grade systemic inflammation (as indicated by CRP levels), and obesity may exert both direct and indirect effects on periodontitis through elevated ferritin levels. Additionally, ferritin is posited to influence periodontitis directly or indirectly by insulin resistance (HOMA-IR). Correlations between CRP and ferritin, as well as insulin resistance with periodontitis were considered.


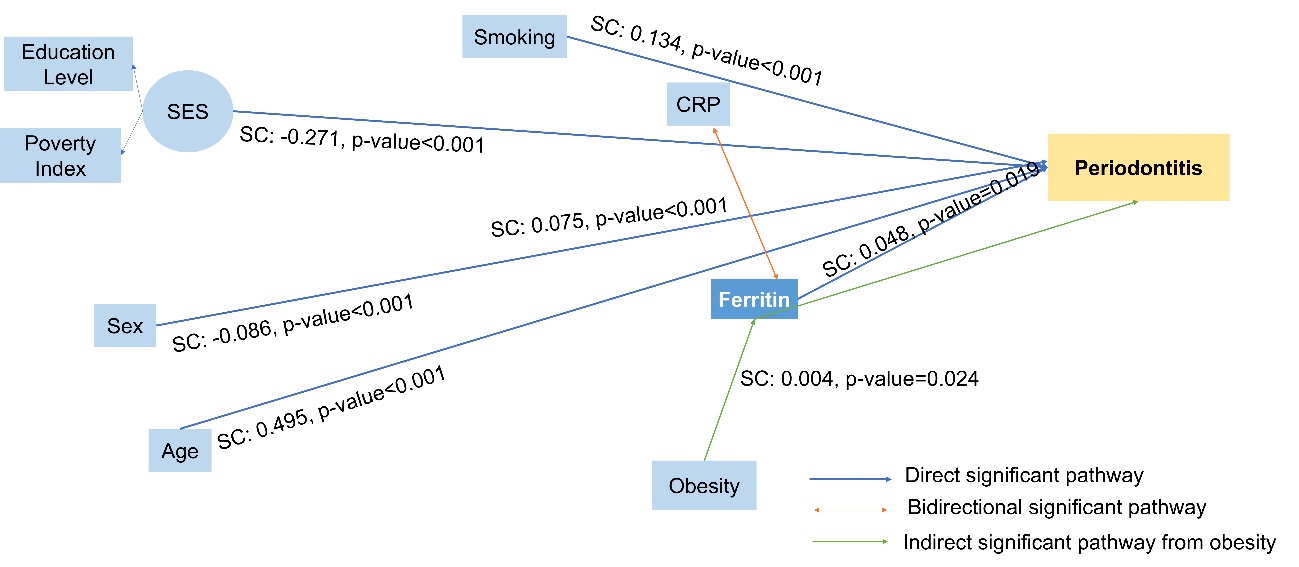


**Figure S2**. Main Results of the model considering correlation between CRP and ferritin, and insulin resistance with periodontitis were considered. High ferritin serum levels remained associated with Periodontitis Stages. CRP and Ferritin were correlated.

**
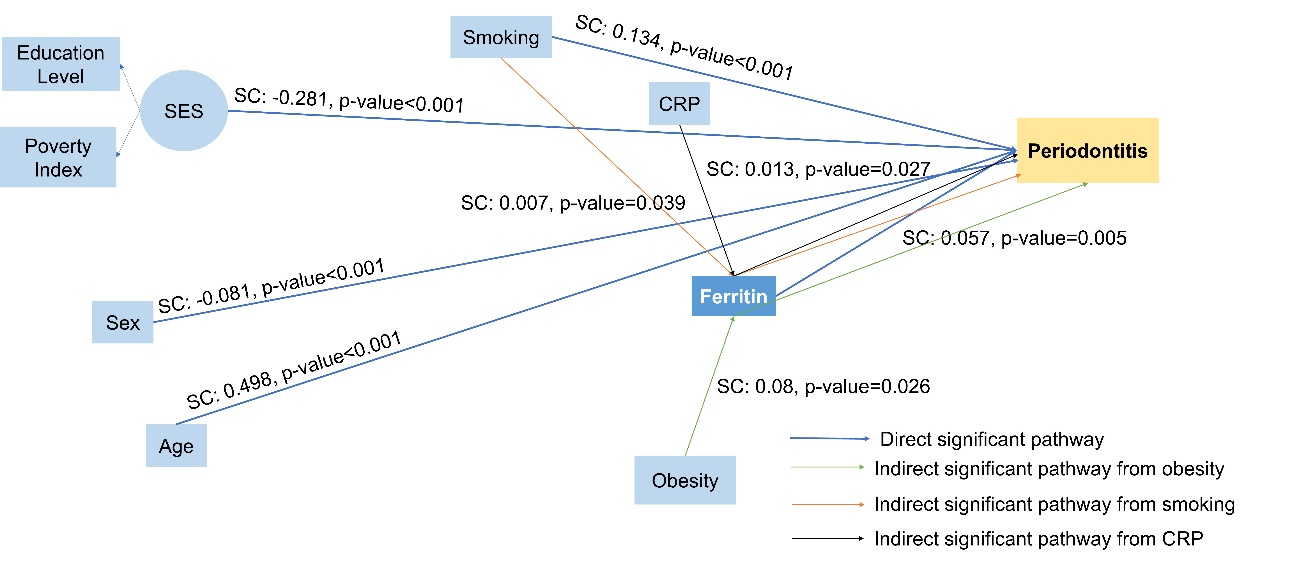
**

**Figure S3.** Main results of the sensibility analysis after exclusion of participants with diabetes, cardiovascular diseases and altered alanine and aspartate serum levels (n=6,620). Ferritin remained associated with Periodontitis Stages. Indirect significant pathways from smoking, low-grade inflammation (CRP) and obesity were also observed.
